# Health and economic benefits of improving pre‐hospital identification of stroke in Australian women: a modelling study

**DOI:** 10.5694/mja2.52701

**Published:** 2025-06-13

**Authors:** Thomas Gadsden, Lei Si, Emily R Atkins, Cheryl Carcel, Xia Wang, Stephen Jan, Mark Woodward, Laura E Downey

**Affiliations:** ^1^ The George Institute for Global Health Australia, UNSW Sydney Sydney NSW; ^2^ Western Sydney University Sydney NSW; ^3^ Translational Health Research Institute, Western Sydney University Sydney NSW; ^4^ The George Institute of Global Health UK, Imperial College London London United Kingdom

**Keywords:** Stroke, Thrombolysis, Healthcare disparities, Health services research, ________________________________________________________________________

## Abstract

**Objective:**

To estimate the long term gains in life years and quality‐adjusted life years (QALYs) and the cost savings that could be achieved if ischaemic stroke was identified in women with the same level of accuracy received by men, versus the status quo.

**Design:**

Decision tree and Markov model decision analysis.

**Settings, participants:**

Two arms including 5513 women aged under 70 years: a hypothetical scenario, in which women receive the same level of accuracy of stroke identification as men (yet experienced symptoms relevant to women); and the status quo. Transitions between post‐stroke health states, recurrent stroke and death were made in 1‐year cycles over 50 years from a societal perspective.

**Main outcome measures:**

Years of life lived, QALYs and costs per patient in the hypothetical scenario relative to the status quo. Results were extrapolated to the national level based on the annual number of ischaemic stroke hospitalisations among women across Australia in the financial year 2020–21.

**Results:**

Compared with the status quo, the hypothetical arm gained 0.14 years of life, gained 0.08 QALYs and saved $2984 per patient. At the national level, for the financial year 2020–21, this equates to 252 life years and 144 QALYs gained, and cost savings of $5.4 million. Outcomes were most sensitive to the probability of an accurate assessment of stroke, short term treatment costs, patient age, and transition probabilities to 90‐day post‐stroke health states.

**Conclusions:**

Enhancing the timely and accurate identification of ischaemic stroke among Australian women in the pre‐hospital setting would yield significant health benefits and cost savings to Australian society as a whole.

**The known:** Stroke is less likely to be identified by emergency medical staff in women compared with men among those who are younger than 70 years in New South Wales, Australia.

**The new:** This modelling study showed that if women received the same level of accuracy in terms of stroke identification as men, they gained life years, gained quality‐adjusted life years and benefited from cost savings per patient from a societal perspective over a lifetime horizon, compared with the status quo.

**The implications:** Younger women experiencing stroke in Australia stand to gain health and economic benefits from more accurate stroke identification in the pre‐hospital setting.

In 2020, premature mortality and lost wellbeing due to stroke was estimated to cost $26 billion in Australia, while the direct financial costs amounted to $6.2 billion.^1^ Stroke among younger adults (ie, those aged < 65 years) has a disproportionately large economic impact by leaving patients disabled in their most productive years.^2^ In recent decades, the incidence of stroke among this population has increased globally and has been framed as a growing public health problem.^3^ While effective treatment is available for acute ischaemic stroke, delays in the pre‐hospital setting are a major barrier to timely care.^4^

Around 80–85% of stroke cases^5^ in Australia are acute ischaemic stroke, for which intravenous thrombolysis (IVT) with tissue plasminogen activator (alteplase) is the current standard treatment for eligible patients and significantly improves the overall likelihood of a good stroke outcome, compared with usual care.^6^ However, the efficacy of IVT is highly time dependent, with treatment eligibility typically limited to within 4.5 hours of stroke onset, and its effectiveness being greater the earlier it is administered.^7^, ^8^ In Australia, where about 73% of patients with stroke were transported by ambulance to hospital in 2023, accurate identification of stroke by emergency medical staff (EMS) is critical to initiate timely treatment.^9^

However, identifying stroke is challenging due to the variable and often non‐specific clinical presentations of stroke patients, as well as high rates of stroke mimics.^10^ Further, recognised sex differences in stroke epidemiology, presentation and risk factors^11^ mean that women are more likely than men to be misdiagnosed by EMS,^12^, ^13^, ^14^, ^15^ potentially leading to treatment delays.^4^, ^16^ Whether these delays reduce women's likelihood of receiving timely IVT treatment is debated. Some studies have reported lower IVT treatment rates among women compared with men,^17^, ^18^, ^19^ while others have found no difference,^20^, ^21^ suggesting that observed sex differences might be attributed to sociodemographic and clinical factors, such as age, initial stroke severity and comorbidities.

Regardless, accurate assessment of stroke by EMS is essential for timely treatment. In Australia, only patients with suspected stroke receive priority transport (ie, blue lights and sirens) to thrombolysis‐capable hospitals, accompanied by pre‐notifications to facilitate rapid treatment.^22^, ^23^ While all ambulance‐transported patients are evaluated on arrival at hospital, patients who are suspected to have had a stroke typically arrive faster and are more likely to receive IVT and receive it sooner.^24^, ^25^, ^26^ Consequently, disparities in stroke identification and timely treatment can translate into significant health and economic loss. In this study, we aimed to explore the potential health and economic benefits of increasing ischaemic stroke identification rates among Australian women to match those experienced by men.

## Methods

### Study design

This modelled analysis was based on a study by Wang and colleagues,^15^ which linked ambulance emergency medical records and hospital admitted patient data to assess the accuracy of stroke identification by EMS in New South Wales, Australia. Among adults younger than 70 years, EMS accurately identified 28.9% of strokes in women (meaning they also had a subsequent clinical stroke diagnosis) compared with 35.0% in men (adjusted odds ratio, 0.89; 95% CI, 0.82–0.97). Close to 90% of the strokes assessed in the cohort described by Wang and colleagues^15^ were ischaemic in nature. In this analysis, we used a simplified assumption that all strokes in the cohort were ischaemic in nature and therefore would likely benefit from timely IVT intervention should it be clinically indicated and available.

Sex was recorded using binary male/female categories extracted from medical records and reflects biological classification. These data were not self‐reported. References to “sex” in this study align with this classification.

### Model overview and cohort

We used a short term decision tree model (3‐month time horizon) combined with a long‐term Markov state‐transition model (50 years) with annual cycles, designed in TreeAge Pro (TreeAge Software) (Supporting Information, figure 1) to compare the costs and effectiveness between two arms: a hypothetical scenario, in which women receive the same level of accuracy of stroke identification by EMS as men (ie, 35.0% accuracy); and the status quo (ie, 28.9% and 35.0% accuracy for women and men, respectively). The model cohort was derived from the study by Wang and colleagues^15^ and consisted of 5513 women (mean age, 57.8 years [SD, 10.9 years]). We report our analysis according to the Consolidated Health Economic Evaluation Reporting Standards 2022 checklist (Supporting Information, table 1).^27^

### Model structure

In the first 3 months, patients enter the model after having an ischaemic stroke, which may be accurately or inaccurately identified by EMS in the pre‐hospital setting. Given that stroke misidentification can delay hospital arrival and treatment, we assumed that only patients who had an accurately identified case of stroke in the pre‐hospital setting would have the opportunity to receive IVT within the target 60‐minute window.^28^ Thus, patients with accurate stroke identification could receive usual care or IVT (with the possibility of an adverse event), after which they transitioned to one of seven possible health states according to degree of disability as assessed by the modified Rankin Scale (mRS). Patients with inaccurate stroke identification received usual care, which typically includes aspirin, a statin and an antihypertensive medication when indicated, and then transitioned to one of the seven possible mRS health states.

Patients who survived (mRS 0–5) at the end of the first 3 months would enter the Markov state‐transition model, which we used to evaluate costs and health outcomes in a lifetime horizon (ie, 50 years). In each 12‐month cycle, patients could experience one of the following: recurrent stroke resulting in death or transition to a worse health state; death from background mortality; or no event (ie, remain in the same health state).

### Model inputs

#### Transition probabilities

The inputs parameters for the model, which were obtained from recently published literature, are summarised in Box 1. The initial probabilities of accurate stroke identification were sourced from the study by Wang and colleagues.^15^ The median rate of IVT provision within 60 minutes of hospital arrival for ischaemic stroke cases in NSW was derived from the Stroke Foundation's 2023 National Stroke Audit.^9^ The probability of an adverse event following IVT was sourced from a retrospective analysis of 9238 ischaemic stroke patients treated with IVT between 2018 and 2021.^29^ Transition probabilities to 90‐day mRS scores following an adverse event were sourced from a cohort study of 985 ischaemic stroke patients treated with IVT in Finland between 1995 and 2008.^33^ For the IVT arm without adverse events, 90‐day mRS transition probabilities were obtained from the control arm of the ENCHANTED trial for participants younger than 70 years, by sex.^32^ This international trial tested intensive versus guideline‐recommended blood pressure lowering treatment for thrombolysis‐eligible patients. For patients who received usual care alone, 90‐day mRS transition probabilities were obtained from the AVERT trial, which examined outcomes for patients who received very early mobilisation in addition to usual care.^31^

Box 1 **Input parameters**

**Table:** 

Parameter	Base case value	Interval	Distribution	Source
**Year 1**				
Probability stroke is accurately identified in women	0.29	0.28–0.30	Triangular	15
Probability stroke is accurately identified in men	0.35	0.34–0.36	Triangular	15
Probability of receiving thrombolysis within 60 minutes of hospital arrival	0.41	NA	NA	9
Probability of an adverse event following thrombolysis	0.02	NA	NA	29
All‐cause mortality at 12 months	0.12	0.11–0.12	Triangular	30
Probability of recurrent stroke at 12 months	0.10	0.09–0.12	Triangular	30
Health state utility by mRS				
mRS 0	0.85	0.76–1.00	Triangular	31
mRS 1	0.78	0.67–0.94	Triangular	31
mRS 2	0.67	0.53–0.89	Triangular	31
mRS 3	0.30	0.12–0.42	Triangular	31
mRS 4	0.11	0.02–0.20	Triangular	31
mRS 5	0.03	0.00–0.07	Triangular	31
90‐day transition probability following thrombolysis by mRS				
mRS 0	0.32	0.29–0.36	Triangular	32
mRS 1	0.24	0.21–0.27	Triangular	32
mRS 2	0.16	0.14–0.19	Triangular	32
mRS 3	0.11	0.09–0.14	Triangular	32
mRS 4	0.08	0.06–0.10	Triangular	32
mRS 5	0.03	0.02–0.04	Triangular	32
mRS 6	0.06	0.04–0.07	Triangular	32
90‐day transition probability following adverse event by mRS				
mRS 0	0	NA	NA	33
mRS 1	0	NA	NA	33
mRS 2	0.09	NA	NA	33
mRS 3	0.05	NA	NA	33
mRS 4	0.05	NA	NA	33
mRS 5	0.19	NA	NA	33
mRS 6	0.62	NA	NA	33
90‐day transition probability following usual care by mRS				
mRS 0	0.09	0.07–0.10	Triangular	31
mRS 1	0.19	0.17–0.22	Triangular	
mRS 2	0.18	0.16–0.21	Triangular	31
mRS 3	0.23	0.20–0.25	Triangular	31
mRS 4	0.13	0.11–0.16	Triangular	31
mRS 5	0.09	0.06–0.11	Triangular	31
mRS 6	0.08	0.06–0.11	Triangular	31
**After year 1**				
Health state utility by mRS				
mRS 0	0.85	0.80–1.00	Triangular	34
mRS 1	0.80	0.75–0.90	Triangular	34
mRS 2	0.70	0.53–0.75	Triangular	34
mRS 3	0.51	0.45–0.65	Triangular	34
mRS 4	0.30	0.25–0.55	Triangular	34
mRS 5	0.15	0.00–0.32	Triangular	34
Probability of recurrent stroke	0.02	0.02–0.02	Triangular	30

mRS = modified Rankin Scale; mRS 0 = no symptoms; mRS 1 = no significant disability; mRS 2 = slight disability; mRS 3 = moderate disability; mRS 4 = moderate to severe disability; mRS 5 = severe disability; mRS 6 = dead; NA = not applicable.

The probability of recurrent stroke and all‐cause mortality at 1 year for patients aged 18–64 was sourced from a study that examined long term outcomes following stroke in Australia and New Zealand between 2008 and 2017.^30^ A mortality rate of 8.66% was applied following recurrent stroke, as reported by the Australian Institute of Health and Welfare for those younger than 65 years.^34^, ^35^ Among survivors of recurrent stroke, the probability of transitioning to a worse mRS health state, excluding death, was assumed to be equal. Background mortality was derived from the Australian general population mortality rate using age‐dependent and sex‐dependent death rates from the period 2019–2021.^36^

#### Costs

The cost inputs for the model are shown in Box 2. These medical, non‐medical and indirect costs associated with stroke treatment, categorised by mRS health state, were sourced from a study by Tan and colleagues that modelled the economic and health burden of stroke among younger adults in Australia.^34^ Short term costs were accrued in year 1 of the model, reflecting the high cost of acute hospital care, and long term costs were accrued in all subsequent years. Indirect costs were only accrued by women younger than 64 years, as only 11% of women older than 65 years are engaged in the labour force.^37^ The cost of IVT was added to the short term costs for patients who received IVT as the majority of the AVERT study cohort did not receive this treatment.^31^ The cost of a recurrent stroke was based on findings from an Australian study that assessed the cost‐effectiveness of tenecteplase versus alteplase.^38^ All costs were converted to and are reported in 2022 Australian dollars, and future costs and outcomes were discounted at a rate of 5% per annum.^39^ Ambulance costs are equal in both arms of the model and therefore were not included.

Box 2 **Cost inputs in year 1 and all subsequent years by health state (in 2022 Australian dollars) sourced from study by Tan and colleagues^34^**

**Table:** 

Health state	Year 1			After year 1		
	Medical (interval*)	Non‐medical (interval*)	Indirect (interval*)	Medical (interval*)	Non‐medical (interval*)	Indirect (interval*)
mRS 0	$17 375 ($6387–$29 799)	$640 ($0–$1281)	$16 553 ($0–$36 174)	$1573 ($0–$3146)	$647 ($0–$1294)	—
mRS 1	$24 607 ($6860–$44 216)	$2373 ($0–$4746)	$30 786 ($0–$56 845)	$1573 ($0–$3146)	$647 ($0–$1294)	$3368 ($0–$6737)
mRS 2	$45 401 ($16 670–$57 816)	$5883 ($157–$7258)	$53 408 ($0–$98 187)	$1993 ($0–$3986)	$820 ($0–$1640)	$22 308 ($0–$44 617)
mRS 3	$76 626 ($49 744–$77 473)	$34 269 ($14 756–$45 917)	$40 849 ($0–$98 187)	$1993 ($0–$3986)	$820 ($0–$1640)	$31 319 ($0–$62 639)
mRS 4	$96 153 ($80 229–$116 819)	$65 806 ($32 967–$83 829)	$48 652 ($0–$103 355)	$15 410 ($0–$30,819)	$6346 ($0‐12 692)	$48 764 ($0–$97 303)
mRS 5	$157 399 ($80 295–$181 813)	$93 225 ($52 065–$78 688)	$45 820 ($0–$98 187)	$19 713 ($0–$39 423)	$8118 ($0–$16 236)	$45 820 ($0–$91 640)
mRS 6	$49 037 ($4402–$47 593)	$22 177 ($0–$2952)	$44 507 ($0–$93 019)	—	—	—

mRS = modified Rankin Scale; mRS 0 = no symptoms; mRS 1 = no significant disability; mRS 2 = slight disability; mRS 3 = moderate disability; mRS 4 = moderate to severe disability; mRS 5 = severe disability; mRS 6 = dead. * The interval used for sensitivity analysis.

### Health outcomes

Health‐related quality‐of‐life values (utility scores) were assigned to all health states. The number of years of life lived in each health state was multiplied by the utility score for each health state to calculate quality‐adjusted life years (QALYs). Utility scores were sourced from the study by Tan and colleagues and included as triangular distributions.^34^ Utility scores for the first 12‐month cycle were based on the AVERT study, which used the Assessment of Quality of Life 4D instrument using Australian population preferences.^40^ Long term utility scores by mRS scores were sourced from the published literature.^41^

### Model outcomes

The primary outcomes of the model were the differences in years of life lived, QALYs and costs for the hypothetical arm compared with the status quo arm from a societal perspective. Base case results were extrapolated to the model cohort (*n* = 5513) in each group and to the national level by multiplying outcomes by the annual number of ischaemic stroke hospitalisations in which patients were likely to receive IVT within 60 minutes of hospital arrival. In the financial year 2020–21, the Australian Institute of Health and Welfare reported 7471 stroke hospitalisations for women aged 18 to 64 years.^42^ Based on 2022 data from the Australian Stroke Clinical Registry, which found that 83% of strokes in this group were ischaemic (excluding transient ischaemic attacks), we assumed a similar proportion applied, equating to an estimated 6201 ischaemic strokes.^5^ Nationally, the rate of IVT provision within 60 minutes of hospital arrival was 29% in 2023.^9^ Thus, we multiplied our outcomes by 1798 (ie, the number of hospitalised women aged 25–64 years who had ischaemic stroke and were likely to receive IVT within 60 minutes of hospital arrival).

### Sensitivity analysis

We tested the robustness of our model predictions in one‐way sensitivity analyses in which the model parameters related to transition probabilities, costs and utilities were replaced by their upper or lower 95% confidence interval values. Uncertainty intervals were sourced from relevant publications and, where not available, calculated by deducting and adding 50% or 20% to mean cost and utility parameters, respectively. We also tested the robustness of our model projections in a probabilistic sensitivity analysis using Monte Carlo simulations, in which all model inputs were randomly drawn 10 000 times from distributions of the model inputs, with half‐cycle correction applied.

### Ethics approval

Ethics approval was not required for this study as no individual patient data were used.

## Results

### Base case

Over a lifetime horizon (50 years), the hypothetical group of women accrued 19.56 life years, 11.70 QALYs and $604 784 in costs per person. Under the status quo, the women accrued 19.42 life years, 11.62 QALYs and $607 768 in costs per person. Thus, the hypothetical group gained 0.14 life years, gained 0.08 QALYs and saved $2984 per person. Applied to the total cohort of 5513 women, this equates to 772 additional years of life, 441 additional QALYs, and savings of $16.5 million. When extrapolated to the national cohort of 1798 women aged 25–64 years who were hospitalised for ischaemic stroke and likely to receive IVT within 60 minutes in the financial year 2020–21, this results in 252 additional years of life, 144 additional QALYs, and $5.4 million in cost savings. The outcomes are summarised in Box 3.

Box 3 **Base case results by treatment arm**

**Table:** 

	Life years	QALYs	Costs
Total cohort (*n* = 5513)			
Hypothetical arm	107 834	64 502	$3 334 174 192
Status quo arm	107 062	64 061	$3 350 624 984
Difference	772	441	$5 365 232
Per person			
Hypothetical arm	19.56	11.70	$604 784
Status quo arm	19.42	11.62	$607 768
Difference	0.14	0.08	$2984

QALYs = quality‐adjusted life years.

### Sensitivity analysis

The results of the one‐way sensitivity analyses are illustrated in separate tornado diagrams for incremental costs (Box 4) and incremental effectiveness (Box 5). Incremental costs were most influenced by long term treatment costs (notably for mRS 3 and 4 health states), 90‐day transition probabilities to mRS state 4 (for both IVT and usual care), the probability of accurate stroke identification by EMS and the age of the patient. Incremental effectiveness was most affected by the age of the patient, probability of an accurate stroke assessment and 90‐day transition probabilities to mRS states. Other model parameters, such as the probability and cost of recurrent stroke, adverse events and health state utilities, had less impact on the base case results.

Box 4 Incremental cost per variable*

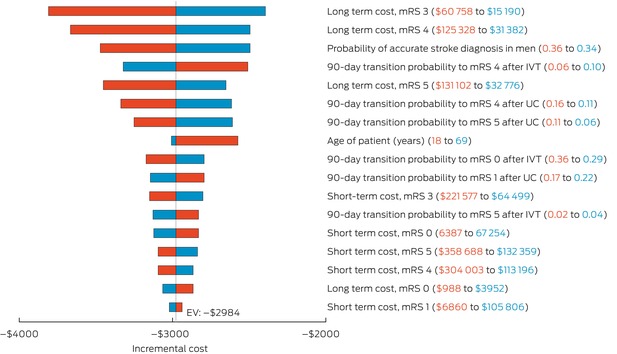

EV = expected value; IVT = intravenous thrombolysis; mRS = modified Rankin scale; UC = usual care. * Red and blue values shown in parentheses represent the lower and upper bounds of each parameter's sensitivity range. Bars indicate the influence of each parameter on incremental costs, with all others held at base case values.

Box 5 Incremental effectiveness by variable*

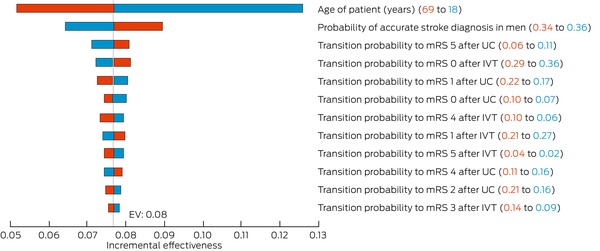

EV = expected value; IVT = intravenous thrombolysis; mRS = modified Rankin scale; UC = usual care. * Red and blue values shown in parentheses represent the lower and upper bounds of each parameter's sensitivity range. Bars indicate the influence of each parameter on incremental QALYs, with all others held at base case values.

In the probabilistic sensitivity analysis, which included 10 000 iterations of all distributions, the hypothetical arm had a 100% probability of being the optimal strategy. A summary of the probabilistic sensitivity analysis results for life years, QALYs and costs for each group is provided in Box 6.

Box 6 **Probabilistic sensitivity analysis results by treatment arm**

**Table:** 

	Life years (95% CI)	QALYs (95% CI)	Costs (95% CI)
Hypothetical arm per person	19.30 (19.21–19.39)	11.52 (11.46–11.58)	$583 194 ($541 790–$546 190)
Status quo arm per person	19.16 (19.07–19.25)	11.44 (11.39–11.50)	$586 042 ($544 689–$549 110)

QALYs = quality‐adjusted life years.

## Discussion

To our knowledge, this is the first study to quantify the potential health and economic gains from addressing sex‐based differences in stroke care. We show that if ischaemic stroke was identified in younger Australian women by EMS at the same rate as in men, these women would experience gains in life years, gains in QALYs and cost savings. While these outcomes are modest on a per‐person basis, they translate into substantial annual societal benefits when extrapolated to the broader Australian population hospitalised for ischaemic stroke and likely to receive IVT. Our findings are consistent with recent estimates of the high costs faced by younger stroke patients;^2^ however, our results also highlight the inequitable health and economic disparities experienced by Australian women in comparison to men. This underscores the critical need for improved identification of stroke by EMS and timely treatment.

The premise of this study is grounded in two streams of evidence: first, accurate stroke identification by EMS leads to faster hospital arrival and timely treatment with thrombolytic therapy;^24^, ^25^ and second, women younger than 70 years are statistically less likely to be recognised as experiencing a stroke in the pre‐hospital setting compared with men.^15^ Notably, the difference in stroke recognition rates between men and women is small, indicating that both sexes are receiving suboptimal care relative to national benchmarks and targets. The national benchmark for thrombolysis within 60 minutes of hospital arrival is 66%,^9^ with a target median onset‐to‐thrombolysis time of under 60 minutes.^43^ Currently, however, there is stark geographic inequity in IVT access across Australia. The national rate of IVT within 60 minutes is 29%, but this drops to zero in Tasmania and the Northern Territory, and the national median onset‐to‐thrombolysis time is 74 minutes.^2^, ^9^ As a result, many Australians experiencing stroke are not receiving optimal standard of care, and sex inequity appears to further amplify this inequity.

Our findings highlight that addressing the small but significant sex gap in early stroke diagnoses could yield important health and economic gains. When extrapolated to the national level, these results reveal the substantial costs to the Australian population of neglecting young women who are experiencing stroke. Building this evidence base is crucial for informing federal and state investment decisions in women's health, an area often characterised by data scarcity and comparatively poorer health outcomes. Although there are methodological limitations to our calculations, such as the underutilisation of services by vulnerable populations,^44^ our estimated annual cost savings of $5.4 million may be conservative given the societal value of healthy, productive women. Increasingly, research is showing that investing in women's health can enhance long term national productivity and yield significant health, economic, social and environmental benefits.^45^ Women's health is often conflated with reproductive health, grossly neglecting the study of underlying sex‐ and gender‐specific risk factors associated with leading causes of disease in women — non‐communicable diseases such as cardiovascular disease and stroke.^46^ Further efforts to study the gendered impacts of non‐communicable diseases and quantify the societal cost of the gender gap in health access and outcomes could drive more targeted policy interventions to protect and enhance the health of women worldwide.

Our findings underscore the need for greater investment in pre‐hospital stroke care systems, particularly in terms of recognising “atypical” symptomatic presentations, where “typical” most often relates to a reference population of men. Educational interventions have shown promise in improving the accuracy of stroke screening and increasing timely treatment with thrombolysis,^47^ while initiatives such as mobile stroke units^48^ and telemedicine solutions^49^ are also advancing acute stroke care. However, to address sex differences in stroke recognition and treatment, further research is needed to explore differences in symptom reporting between women and men, potential provider biases, and the role of sex‐specific guidelines.^50^ As others have recommended, greater attention should be paid to improving diagnosis through standardised diagnostic approaches, implementing strategies to reduce sex differences in investigations and care and routinely inquiring about sex‐specific stroke risk factors.^51^

### Strengths and limitations

A strength of this study is the use of clinical and cost data from an Australian societal perspective^15^, ^34^ and its novel use in quantifying a known sex health gap. However, several limitations must be acknowledged. The most significant is the lack of sex‐disaggregated data for all but one of our key parameters. Consequently, mRS distributions for the usual care arm were derived from the ENCHANTED trial, which included men and women of all ages, predominantly from Asian countries.^32^ Furthermore, obtaining data for women younger than 70 years posed challenges, leading us to use data for 18–64‐year‐olds, which did not align precisely with our cohort. Second, the rate of IVT provision in NSW that we used was higher than the national average, which may limit the generalisability of our findings to the national level. Nevertheless, NSW represents a substantial proportion of the national stroke burden, particularly for the under 70 years age group that we modelled, and provides a valuable benchmark for understanding potential health and economic impacts of accurate pre‐hospital stroke identification and timely intervention. Last, the use of triangular distributions for sensitivity analyses is suboptimal for capturing the full range of uncertainty in model parameters. Despite this, we used confidence intervals to inform the triangular distributions, which was adequate for estimating potential outcomes related to our hypothetical policy question.

### Conclusion

The sex gap in accurate stroke detection in the pre‐hospital setting between men and women disadvantages younger women and costs the Australian population socially and economically. Our findings show that if emergency medical services accurately identified ischaemic stroke in younger Australian women at the same rate as is currently achieved in men, these women could experience longer, healthier lives and significant cost savings. Such health and economic benefits would offer substantial value to Australian society.

## Open access

Open access publishing facilitated by Western Sydney University, as part of the Wiley ‐ Western Sydney University agreement via the Council of Australian University Librarians.

## Competing interests

No relevant disclosures.

## Data sharing

The datasets used and/or analysed during the current study are available from the corresponding author on reasonable request.

## Author contributions

Gadsden T: Methodology; software; formal analysis; writing – original draft; writing – review and editing. Si L: Conceptualization; methodology; software; formal analysis; writing – review and editing; supervision. Atkins ER: Methodology; data curation; writing – review and editing. Carcel C: Conceptualization; writing – review and editing; supervision. Wang X: Data curation; writing – review and editing. Jan S: Writing – review and editing; supervision. Woodward M: Formal analysis; writing – review and editing. Downey LE: Conceptualization; methodology; writing – review and editing; supervision.

Received 15 May 2024, accepted 23 December 2024

## Supporting information


Supplementary figure and table

## References

[1] Deloitte Access Economics . The economic impact of stroke in Australia, 2020. Sydney: Stroke Foundation, 2020. https://strokefoundation.org.au/media/nydptqxi/economic‐impact‐of‐stroke‐report‐30‐october‐final‐report.pdf (viewed May 2023).

[2] Kim J , Neville E , Dalli L , et al. Economic impact of stroke report 2024. Melbourne: Stroke Foundation, 2024. https://strokefoundation.org.au/what‐we‐do/research/economic‐impact‐of‐stroke‐in‐australia (viewed Nov 2024).

[3] Boot E , Ekker MS , Putaala J , et al. Ischaemic stroke in young adults: a global perspective. J Neurol Neurosurg Psychiatry 2020; 91: 411‐417. 32015089 10.1136/jnnp-2019-322424

[4] Sharobeam A , Jones B , Walton‐Sonda D , Lueck CJ . Factors delaying intravenous thrombolytic therapy in acute ischaemic stroke: a systematic review of the literature. J Neurol 2021; 268: 2723‐2734. 32206899 10.1007/s00415-020-09803-6

[5] Cadilhac D , Dalli L , Morrison J , et al. Australian Stroke Clinical Registry annual report 2022. Melbourne: Florey Institute of Neuroscience and Mental Health, 2023. https://auscr.com.au/about/annual‐reports (viewed Jan 2024).

[6] Mead GE , Sposato LA , Sampaio Silva G , et al. A systematic review and synthesis of global stroke guidelines on behalf of the World Stroke Organization. Int J Stroke 2023; 18: 499‐531. 36725717 10.1177/17474930231156753 PMC10196933

[7] Wardlaw JM , Murray V , Berge E , del Zoppo GJ . Thrombolysis for acute ischaemic stroke. Cochrane Database Syst Rev 2014; (7): CD000213. 10.1002/14651858.CD000213.pub3 PMC4153726 25072528

[8] Emberson J , Lees KR , Lyden P , et al. Effect of treatment delay, age, and stroke severity on the effects of intravenous thrombolysis with alteplase for acute ischaemic stroke: a meta‐analysis of individual patient data from randomised trials. Lancet 2014; 384: 1929‐1935. 25106063 10.1016/S0140-6736(14)60584-5 PMC4441266

[9] Stroke Foundation . National stroke audit – acute services report 2023. Melbourne: Stroke Foundation, 2023. https://informme.org.au/stroke‐data/acute‐audits (viewed Feb 2024).

[10] Jones SP , Bray JE , Gibson JM , et al. Characteristics of patients who had a stroke not initially identified during emergency prehospital assessment: a systematic review. Emerg Med J 2021; 38: 387‐393. 33608393 10.1136/emermed-2020-209607 PMC8077214

[11] Carcel C , Woodward M , Wang X , et al. Sex matters in stroke: a review of recent evidence on the differences between women and men. Front Neuroendocrinol 2020; 59: 100870. 32882229 10.1016/j.yfrne.2020.100870

[12] Mould‐Millman NK , Meese H , Alattas I , et al. Accuracy of prehospital identification of stroke in a large stroke belt municipality. Prehosp Emerg Care 2018; 22: 734‐742. 29596006 10.1080/10903127.2018.1447620

[13] Govindarajan P , Friedman BT , Delgadillo JQ , et al. Race and sex disparities in prehospital recognition of acute stroke. Acad Emerg Med 2015; 22: 264‐272. 25728356 10.1111/acem.12595 PMC4355063

[14] Eliakundu AL , Cadilhac DA , Kim J , et al. Determining the sensitivity of emergency dispatcher and paramedic diagnosis of stroke: statewide registry linkage study. J Am Coll Emerg Physicians Open 2022; 3: e12750. 35795711 10.1002/emp2.12750 PMC9249375

[15] Wang X , Carcel C , Hsu B , et al. Differences in the pre‐hospital management of women and men with stroke by emergency medical services in New South Wales. Med J Aust 2022; 217: 143‐148. https://www.mja.com.au/journal/2022/217/3/differences‐pre‐hospital‐management‐women‐and‐men‐stroke‐emergency‐medical 35831059 10.5694/mja2.51652 PMC9541458

[16] Sheppard JP , Mellor RM , Greenfield S , et al. The association between prehospital care and in‐hospital treatment decisions in acute stroke: a cohort study. Emerg Med J 2015; 32: 93‐99. 24099829 10.1136/emermed-2013-203026 PMC4316848

[17] Messe SR , Khatri P , Reeves MJ , et al. Why are acute ischemic stroke patients not receiving IV tPA? Results from a national registry. Neurology 2016; 87: 1565‐1574. 27629092 10.1212/WNL.0000000000003198 PMC5067546

[18] Asdaghi N , Romano JG , Wang K , et al. Sex disparities in ischemic stroke care: FL‐PR CReSD study (Florida‐Puerto Rico Collaboration to Reduce Stroke Disparities). Stroke 2016; 47: 2618‐2626. 27553032 10.1161/STROKEAHA.116.013059 PMC5039084

[19] Strong B , Lisabeth LD , Reeves M . Sex differences in IV thrombolysis treatment for acute ischemic stroke: a systematic review and meta‐analysis. Neurology 2020; 95: e11‐e22. 32522796 10.1212/WNL.0000000000009733

[20] Bonkhoff AK , Karch A , Weber R , et al. Female stroke: sex differences in acute treatment and early outcomes of acute ischemic stroke. Stroke 2021; 52: 406‐415. 33493053 10.1161/STROKEAHA.120.032850

[21] Marko M , Miksova D , Haidegger M , et al. Trends in sex differences of functional outcome after intravenous thrombolysis in patients with acute ischemic stroke. Int J Stroke 2024; 19: 1147‐1154. 39086256 10.1177/17474930241273696

[22] Australian Commission on Safety and Quality in Health Care . Acute Stroke Clinical Care Standard. Sydney: ACSQHC, 2019. https://www.safetyandquality.gov.au/standards/clinical‐care‐standards/acute‐stroke‐clinical‐care‐standard (viewed Nov 2024).

[23] Nielsen VM , DeJoie‐Stanton C , Song G , et al. The association between presentation by EMS and EMS prenotification with receipt of intravenous tissue‐type plasminogen activator in a state implementing stroke systems of care. Prehosp Emerg Care 2020; 24: 319‐325. 31490714 10.1080/10903127.2019.1662862 PMC7113086

[24] Magnusson C , Herlitz J , Sunnerhagen KS , et al. Prehospital recognition of stroke is associated with a lower risk of death. Acta Neurologica Scandinavica 2022; 146: 126‐136. 35385136 10.1111/ane.13618 PMC9546484

[25] Richards CT , Sucharew H , Kissela BM , et al. Prehospital identification of acute ischemic stroke is associated with faster and more frequent thrombolysis. Stroke 2021; 52 (Suppl 1): abstract 19.

[26] Abboud ME , Band R , Jia J , et al. Recognition of stroke by EMS is associated with improvement in emergency department quality measures. Prehosp Emerg Care 2016; 20: 729‐736. 27246289 10.1080/10903127.2016.1182602

[27] Husereau D , Drummond M , Augustovski F , et al. Consolidated Health Economic Evaluation Reporting Standards 2022 (CHEERS 2022) explanation and elaboration: a report of the ISPOR CHEERS II Good Practices Task Force. Value Health 2022; 25: 10‐31. 35031088 10.1016/j.jval.2021.10.008

[28] Fassbender K , Balucani C , Walter S , et al. Streamlining of prehospital stroke management: the golden hour. Lancet Neurol 2013; 12: 585‐596. 23684084 10.1016/S1474-4422(13)70100-5

[29] Warach SJ , Ranta A , Kim J , et al. Symptomatic intracranial hemorrhage with tenecteplase vs alteplase in patients with acute ischemic stroke: the Comparative Effectiveness of Routine Tenecteplase vs Alteplase in Acute Ischemic Stroke (CERTAIN) Collaboration. JAMA Neurol 2023; 80: 732‐738. 37252708 10.1001/jamaneurol.2023.1449 PMC10230371

[30] Peng Y , Ngo L , Hay K , et al. Long‐term survival, stroke recurrence, and life expectancy after an acute stroke in Australia and New Zealand from 2008–2017: a population‐wide cohort study. Stroke 2022; 53: 2538‐2548. 35418238 10.1161/STROKEAHA.121.038155

[31] AVERT Trial Collaboration group . Efficacy and safety of very early mobilisation within 24 h of stroke onset (AVERT): a randomised controlled trial. Lancet 2015; 386: 46‐55. 25892679 10.1016/S0140-6736(15)60690-0

[32] Anderson CS , Huang Y , Lindley RI , et al. Intensive blood pressure reduction with intravenous thrombolysis therapy for acute ischaemic stroke (ENCHANTED): an international, randomised, open‐label, blinded‐endpoint, phase 3 trial. Lancet 2019; 393: 877‐888. 30739745 10.1016/S0140-6736(19)30038-8

[33] Strbian D , Sairanen T , Meretoja A , et al. Patient outcomes from symptomatic intracerebral hemorrhage after stroke thrombolysis. Neurology 2011; 77: 341‐348. 21715707 10.1212/WNL.0b013e3182267b8c

[34] Tan E , Gao L , Collier JM , et al. The economic and health burden of stroke among younger adults in Australia from a societal perspective. BMC Public Health 2022; 22: 218. 35114974 10.1186/s12889-021-12400-5 PMC8811989

[35] Australian Institute of Health and Welfare . Cardiovascular disease in women (AIHW Cat. No. CDK 15). Canberra: AIHW, 2019. https://www.aihw.gov.au/reports/heart‐stroke‐vascular‐diseases/cardiovascular‐disease‐in‐women‐main/summary (viewed Oct 2023).

[36] Australian Bureau of Statistics . Life tables (reference period 2019–2021). Canberra: ABS, 2022. https://www.abs.gov.au/statistics/people/population/life‐tables/latest‐release (viewed Sept 2023).

[37] Australian Bureau of Statistics . Labour force, Australia, detailed. Canberra: ABS, 2023. https://www.abs.gov.au/statistics/labour/employment‐and‐unemployment/labour‐force‐australia‐detailed/latest‐release (viewed Jan 2024).

[38] Gao L , Moodie M , Mitchell PJ , et al. Cost‐effectiveness of tenecteplase before thrombectomy for ischemic stroke. Stroke 2020; 51: 3681‐3689. 33023423 10.1161/STROKEAHA.120.029666

[39] Medical Services Advisory Committee . Guidelines for preparing assessments for the Medical Services Advisory Committee. Canberra: Australian Government Department of Health and Aged Care, 2021. https://www.msac.gov.au/resources/guidelines‐preparing‐assessments‐msac (viewed May 2023).

[40] Cumming TB , Churilov L , Collier J , et al; AVERT Trial Collaboration Group. Early mobilization and quality of life after stroke: findings from AVERT. Neurology 2019; 93: e717‐e728. 31350296 10.1212/WNL.0000000000007937 PMC6715509

[41] Peultier AC , Pandya A , Sharma R , et al. Cost‐effectiveness of mechanical thrombectomy more than 6 hours after symptom onset among patients with acute ischemic stroke. JAMA Netw Open 2020; 3: e2012476. 32840620 10.1001/jamanetworkopen.2020.12476 PMC7448828

[42] Australian Institute of Health and Welfare . Heart, stroke and vascular disease: Australian facts (AIHW Cat. No. CVD 92). Canberra: AIHW, 2023. https://www.aihw.gov.au/reports/heart‐stroke‐vascular‐diseases/hsvd‐facts (viewed Nov 2023).

[43] Australian Stroke Coalition . National stroke targets 30/60/90. Melbourne: Stroke Foundation, 2024. https://australianstrokecoalition.org.au/projects/targets (viewed Jan 2024).

[44] Bhaskar S , Thomas P , Cheng Q , et al. Trends in acute stroke presentations to an emergency department: implications for specific communities in accessing acute stroke care services. Postgrad Med J 2019; 95: 258‐264. 31097575 10.1136/postgradmedj-2019-136413

[45] Bloom DE , Kuhn M , Prettner K . The contribution of female health to economic development. Econ J 2020; 130: 1650‐1677.

[46] Remme M , Vassall A , Fernando G , Bloom DE . Investing in the health of girls and women: a best buy for sustainable development. BMJ 2020; 369: m1175. 32487585 10.1136/bmj.m1175 PMC7265042

[47] Chowdhury SZ , Baskar PS , Bhaskar S . Effect of prehospital workflow optimization on treatment delays and clinical outcomes in acute ischemic stroke: a systematic review and meta‐analysis. Acad Emerg Med 2021; 28: 781‐801. 33387368 10.1111/acem.14204

[48] Turc G , Hadziahmetovic M , Walter S , et al. Comparison of mobile stroke unit with usual care for acute ischemic stroke management: a systematic review and meta‐analysis. JAMA Neurol 2022; 79: 281‐290. 35129584 10.1001/jamaneurol.2021.5321 PMC8822443

[49] Meyer BC , Raman R , Hemmen T , et al. Efficacy of site‐independent telemedicine in the STRokE DOC trial: a randomised, blinded, prospective study. Lancet Neurol 2008; 7: 787‐795. 18676180 10.1016/S1474-4422(08)70171-6 PMC2744128

[50] DeFilippis EM , Van Spall HGC . Is it time for sex‐specific guidelines for cardiovascular disease? J Am Coll Cardiol 2021; 78: 189‐192. 34238440 10.1016/j.jacc.2021.05.012

[51] Rexrode KM , Madsen TE , Yu AYX , et al. The impact of sex and gender on stroke. Circ Res 2022; 130: 512‐528. 35175851 10.1161/CIRCRESAHA.121.319915 PMC8890686

